# Life in focus

**DOI:** 10.7554/eLife.14169

**Published:** 2016-02-09

**Authors:** Carol Dieckmann, Telsa Mittelmeier

**Affiliations:** Department of Molecular and Cellular Biology, University of Arizona, Tucson, United Statesdieckman@email.arizona.edu; Department of Molecular and Cellular Biology, University of Arizona, Tucson, United States

**Keywords:** Cyanobacteria, Synechocystis sp PCC6803, Thermosynechococcus elongatus, Phototaxis, Signal transduction, Micro-optics, Other

## Abstract

Single-celled photosynthetic bacteria determine the direction of incoming light by acting as lenses.

**Related research article** Schuergers N, Lenn T, Kampmann R, Meissner M, Esteves T, Temerinac-Ott M, Korvink JG, Lowe AR, Mullineaux CW, Wilde A. 2016. Cyanobacteria use micro-optics to sense light direction. *eLife ***5**:e12620. doi: 10.7554/eLife.12620

**Image** Blue-green bacteria move in response to changes in local light conditions

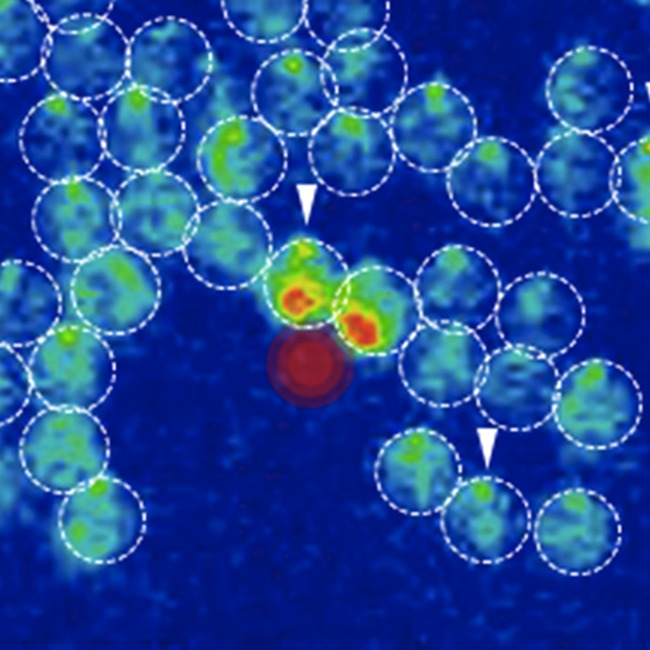

Land plants are restricted to the location in which they germinate and grow, and individual plants are at the mercy of the light conditions where a given seed lands. However, some single-celled photosynthetic organisms exhibit behavior known as “phototaxis” that involves moving in response to local light conditions (Gualteri, 2001). For example, the green alga, *Chlamydomonas reinhardtii*, can swim toward light to increase photosynthesis, but it can also swim away from bright light to avoid damage to molecular complexes required for photosynthesis (Foster and Smyth, 1980).

What are the minimal requirements for phototaxis? First, the organism must have a molecule that detects light. In most algal systems these photoreceptors act as both a light receptor and as an ion channel to trigger downstream signal transduction, similar to the rhodopsins found in bacteria (Nagel et al., 2005). The second requirement for phototaxis is motility. The third requirement is the ability to determine the direction of the incoming light, so that the cell can respond by moving in the appropriate direction.

This final requirement, “knowing where the light is”, has been solved by evolution in a variety of ways in different organisms. Now, in eLife, Conrad Mullineaux, Annegret Wilde and co-workers – including Nils Schuergers as first author – report that in a genus of bacteria called Synechocystis, the cells themselves act as lenses that can focus light (Schuergers et al., 2016). They use a combination of high-end microscopy and materials science to show that light hitting the convex surface of the cell is focused by the refractive properties of the cell body into a spot on the opposite side of the cell.

How does focusing light result in directional movement? To move in a given direction, some kind of asymmetry must be established in the cell. In Synechocystis, motility relies on tiny cell-surface projections called pili that grab the substrate and pull the cell forward, before releasing it and starting another round of grabbing and pulling. Schuergers et al. – who are based in Freiburg, Karlsruhe, London and Porto – found that the cells move away from the focused spot, toward the external light source. The required asymmetry is established by motor proteins that drive assembly of the pili, which cluster on the side of the cell away from the focused spot. The photoreceptors, on the other hand, are evenly distributed in the cell membrane.

By contrast, in other single-celled systems, such as Chlamydomonas and other eukaryotic algae, the flagella responsible for the movement are fixed at one end of the cell by the basal bodies from which they extend (Marshall, 2008). Thus, to provide useful information for directional movement, the photoreceptors must be clustered in one location in the cell, in organelles called eyespots. The position of the eyespot relative to the flagella is fixed by the asymmetric properties of the microtubule cytoskeleton established by the basal bodies (Holmes and Dutcher, 1989; Kamiya and Witman, 1984). Additionally, elaborate light-absorbing structures, often provided by the chloroplast, block light traveling through the cell (Kawai and Kreimer, 2000).

Why did such complicated phototaxis systems evolve in eukaryotic algae? Perhaps having the flagella fixed at one end of primordial cells dictated first the clustering of the photoreceptors, followed by the development of the variety of “light-blocking” systems in different species, which often include structures evolved from the cyanobacterium ancestor engulfed by the host cell.

That cells could act as lenses was proposed previously as an explanation for the phototactic behavior observed in colorless single-celled algae (Sineshchekov et al., 1994). And recently it was shown that the Chlamydomonas-like cells that comprise multicellular Volvox colonies are lenses (Kessler et al., 2015). These cells focus light most acutely a few cell diameters away. Kessler et al. suggested that this light-focusing ability of individual cells influenced the evolutionary path from single cells to the more complex volvocine algae containing thousands of cells.

The work of Schuergers et al. is the first truly elegant demonstration of “lensing” by a bacterial cell. These data are also a powerful reminder of the influence of light on life. Photoreceptors have evolved to capture photons and signal transduction pathways have evolved to allow cells to harness their energy. More pointedly, this work reminds us that light is directional and can be absorbed, reflected and refracted by interactions with living cells. This is a beautiful demonstration of the intersection of physics and biology, not only at the cellular level, but at the experimental level as well.

**Figure 1.**
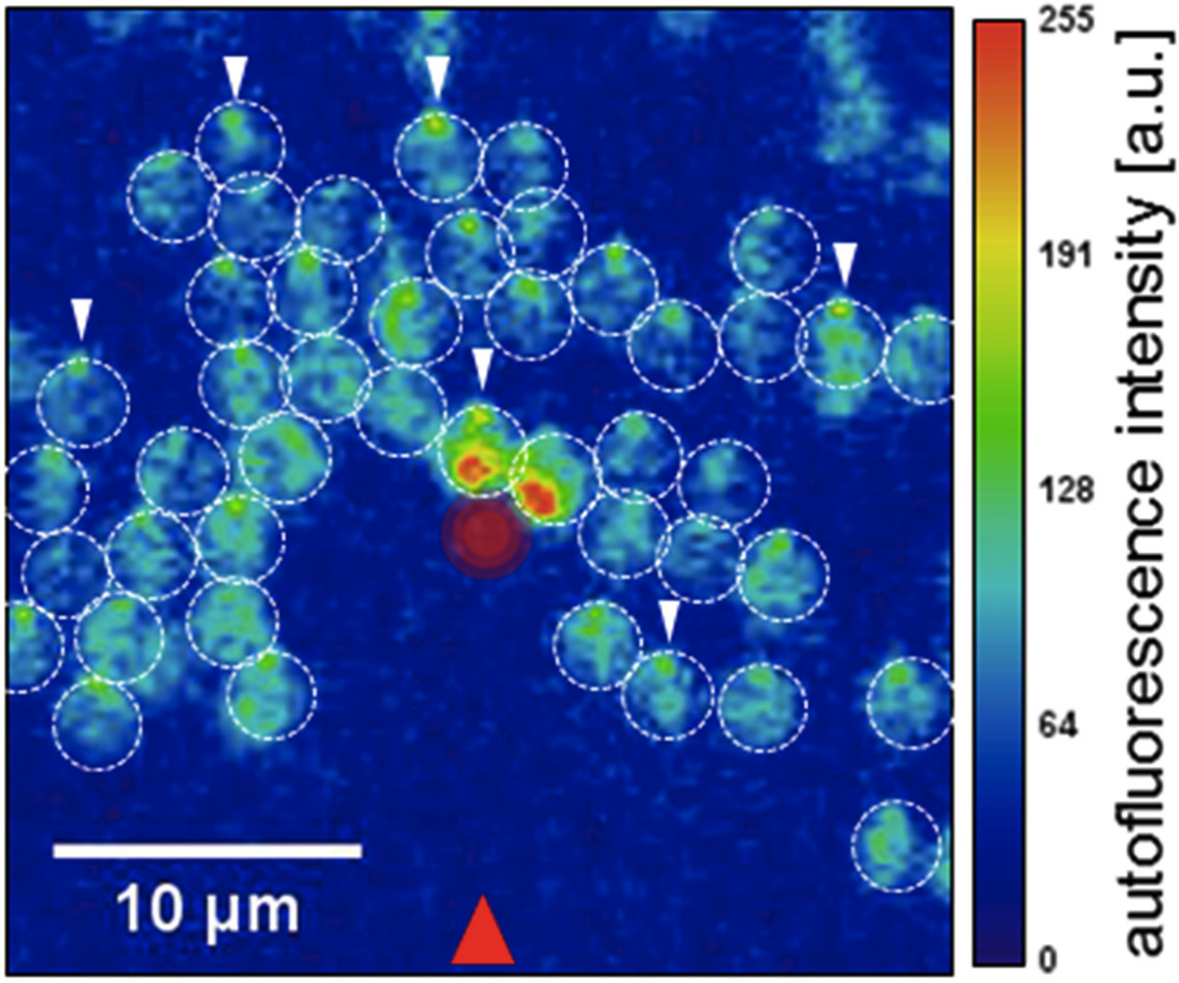
Single-celled photosynthetic bacteria acting as lenses. Schuergers et al. illuminated Synechocystis cells on an agar surface with a light source from the direction marked with a red arrow. Each cell, acting as a lens, focused the light on the rear edge of the cell (white arrows), and moved toward the light. A strong laser light from above (red circle in center), caused cells entering the small laser-illuminated area on the agar to reverse direction. (See figure 4 in Schuergers et al., 2016 for full details).
